# Expression pattern and clinical significance of β-catenin gene and protein in patients with primary malignant and benign bone tumors

**DOI:** 10.1038/s41598-022-13685-1

**Published:** 2022-06-08

**Authors:** Narges Khademian, Alireza Mirzaei, Ameinh Hosseini, Leila Zare, Shima Nazem, Pegah Babaheidarian, Alireza Sheikhi, Zohreh Abdolvahabi, Mostafa Ibrahimi, Khodamorad Jamshidi, Mahtab Rahbar, Vahid Salimi, Masoumeh Tavakoli-Yaraki

**Affiliations:** 1 Department of Biochemistry, School of Medicine, Iran University of Medical Sciences, P.O. Box: 1449614535, Tehran, Iran; 2 Bone and Joint Reconstruction Research Center, Shafa Orthopedic Hospital, Iran University of Medical Sciences, Tehran, Iran; 3 Department of Physiology, Faculty of Medical Sciences, Tarbiat Modares University, Tehran, Iran; 4 Department of Laboratory Medicine, Faculty of Paramedical Sciences, Shahid Beheshti University of Medical Sciences, Tehran, Iran; 5 Department of Pathology, School of Medicine, Iran University of Medical Sciences, Tehran, Iran; 6 Metabolic Diseases Research Center, Research Institute for Prevention of Non-Communicable Disease, Cellular and Molecular Research Center, Qazvin University of Medical Sciences, Qazvin, Iran; 7 Department of Clinical Biochemistry, School of Medicine, Tarbiat Modares University, Tehran, Iran; 8 Department of Virology, School of Public Health, Tehran University of Medical Sciences, Tehran, Iran

**Keywords:** Cancer, Biomarkers

## Abstract

This study is aimed to unravel the status of local and circulating β-catenin in different primary bone tumors and its relevance to tumor types, severity, and chemotherapy. The β-catenin mRNA expression level and the expression of the protein (intensity level) were evaluated in tumor tissue and peripheral blood mononuclear cells of 150 patients with different types of primary bone tumors (78 malignant and 72 benign tumors) using Real-Time PCR and immunohistochemistry. The β-catenin mRNA expression level and the expression of the protein were increased in bone tumors which was positively correlated with the tumor malignancy. Amongst osteosarcoma, Ewing's Sarcoma, chondrosarcoma, osteochondroma, Giant Cell Tumor, and exostosis tumors, the osteosarcoma, and Giant Cell Tumor groups showed the highest level of β-catenin expression. The β-catenin expression in malignant bone tumors was significantly correlated with tumor grade, size, metastasis, tumor recurrent, and the level of response to chemotherapy. A similar pattern of β-catenin gene expression and its association with tumor characteristics was detected in the patient's peripheral blood cells. The simultaneous increase in the expression of the β-catenin gene and protein in tumor tissue and in circulating blood cells and its relationship with tumor severity indicates the possible promoting role of β-catenin in primary bone tumor pathogenesis.

## Introduction

Primary bone tumors are considered as non-frequent mesenchyme-originated solid tumors which affected individuals at all age ranges^1^. The bone tumor incidence rate comprises at least two distinct peaks of an individual's life in the second decade and late adulthood^2^. The dynamic of bone tissue-dependent on the tissue’s physiological and pathological condition which makes it susceptible to cellular re-arrangement, tumor cell formation, and tumor cell implantation^3^. Despite recent improvements regarding primary bone tumor diagnosis and therapeutic strategies, still nonspecific symptoms besides late patient referring and early detection failing are prominent drawbacks that cause cancer-induced morbidity and mortality^4^. The Wnt/β-catenin pathway received considerable attention as effective cellular signaling which is associated with the pathogenesis of several cancers ^5^. Various cellular functions likewise cell proliferation, polarity, fate, migration, renewal, regeneration as well as organogenesis during embryonic development and tissue homeostasis are under the regulation of the canonical Wnt/β-catenin pathway^6^. Beta-catenin is the transcriptional activator that belongs to the cadherin protein complex and controls the expression of several targets involved in cell development and fate. The β-catenin accumulation or mutation was detected in some malignancies likewise hepatocellular carcinoma, endometrial carcinoma, gastric adenocarcinoma^7^ Interestingly, the relevance of canonical Wnt signaling machinery was reported to be involved in bone homeostasis^8^. It was reported that activation of β-catenin in osteocytes enhances the osteoclasts and osteoblasts' activity resulted in bone gain and is required for osteocyte viability and maintaining normal bone mass^9,10^. Additionally, deregulation of β-catenin can be involved in bone–related diseases likewise bone metabolic disease and bone tumors^11^. It was revealed that the Wnt signaling pathway is implicated in Ewing's Sarcoma biology and might be regulated by the EWSR1-FLI1, the major oncogene of Ewing's Sarcoma and involved in tumor migration or differentiation^12,13^. Based on the mechanistic studies regarding osteosarcoma pathogenesis, it seems that the canonical Wnt signaling pathway is involved in osteosarcoma progression possibly through naked cuticle homolog 2 (NKD2) which negatively regulates the Wnt/β-catenin pathway^14^.

Despite promising evidence, questions might be raised due to the status of β-catenin as a pivotal mediator of the Wnt canonical pathway in human samples with diverse bone tumors. How is the expression pattern of β-catenin in prevalent malignant bone tumors? Does the β-catenin expression differ in Ewing's Sarcoma compared to osteosarcoma or chondrosarcoma? How is the β-catenin expression pattern in benign bone tumors? Do chemotherapy treatments affect β –catenin expression in the site of the tumor and peripheral blood? Does the patient's peripheral blood as an invasive source of the patient’s sample reflect the local β-catenin expression in tumors? Is it possible that the patient's peripheral blood serves as a predictor of β-catenin expression pattern in bone cancer? Our study is designed to peruse the abovementioned remarks.

## Results

### The β-catenin gene expression level in different types of primary bone tumors

**Figure 1:**
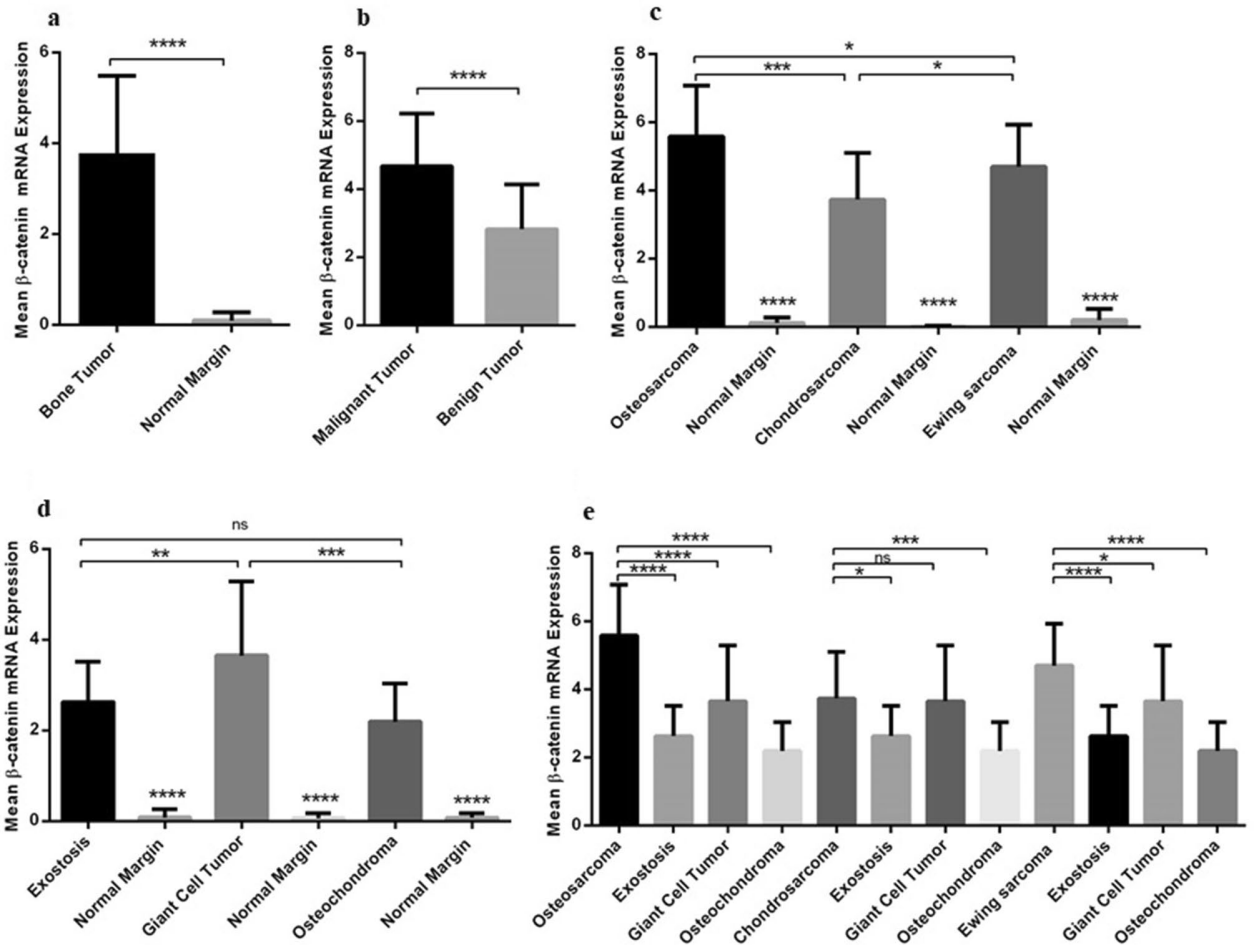
The β-catenin gene expression in primary bone tumors. The β-catenin mRNA expression level was assessed in different bone tumor tissues. The mRNA expression level of β-catenin was increased in a bone tumor (**a**) versus tumor margins also malignant bone tumors versus benign tumors (**b**). An increase in the mRNA expression level of β-catenin was detected in osteosarcoma, Ewing's Sarcoma and chondrosarcoma compared to the matched normal margins (**c**). The β-catenin expression level was increased in benign tumor subtypes including osteochondroma, Giant-cell tumor, and exostosis compared to their matched normal margins (**d**). The β-catenin mRNA expression level was increased in osteosarcoma, chondrosarcoma and Ewing's Sarcoma tumors compared to benign tumor subtypes subsequently (**e**). The comparative CT (2^−ΔCt^) method was applied for gene expression analysis. Based on the normal distribution of data using Kolmogorov–Smirnov analysis, t-student test was used to evaluate the difference in the expression level of β-catenin between groups that are tested in sections a, b, c, d and e. The statistical differences between groups are shown as asterisk (*P < 0.05, **P < 0.01, ***P < 0.001, ****P < 0.0001).

**Figure 2:**
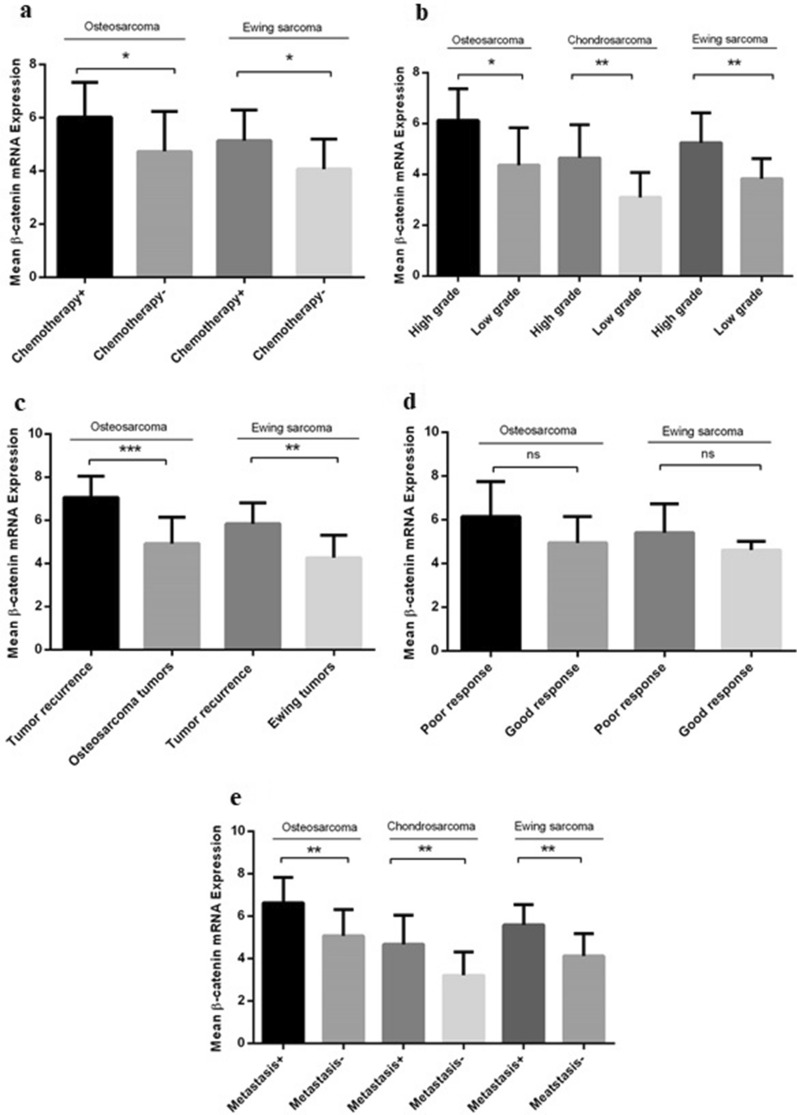
The β-catenin gene expression in malignant bone tumors with different tumor grades, metastasis, recurrent and chemotherapy status. The β-catenin mRNA expression level was evaluated in different types of osteosarcoma, chondrosarcoma, and Ewing's Sarcoma. An increase in the mRNA expression level of β-catenin was observed in chemotherapy-received osteosarcoma and Ewing's Sarcoma tumors (**a**), osteosarcoma, chondrosarcoma, and Ewing's Sarcoma high-grade tumors (**b**), recurrent osteosarcoma and Ewing's Sarcoma tumors (**c**), osteosarcoma and Ewing's Sarcoma tumors with poor response to the chemotherapy (**d**), and osteosarcoma, chondrosarcoma, and Ewing's Sarcoma metastatic tumors in each malignant group (**e**). The comparative CT (2^−ΔCt^) method was applied for gene expression analysis. Kolmogorov–Smirnov analysis showed normal distribution of data therefore t-student test was used to evaluate the difference in the expression level of β-catenin between groups. The statistical differences between groups are shown as asterisk (*P < 0.05, **P < 0.01, ***P < 0.001).

Our data indicated that the β-catenin expression increases significantly in bone tumors compared to normal bone tissues (P < 0.0001) which is shown in Fig. 1a. The mean and standard error mean (SEM) of β-catenin mRNA level in tumor and tumor margin groups was 3.782 ± 0.1396 and 0.09516 ± 0.01535, respectively. In comparing the β-catenin expression status between malignant and benign bone tissues, it was revealed that the β-catenin mRNA expression increased significantly in malignant bone tumors (4.668 ± 0.1755) compared to benign bone tumors (2.823 ± 0.1551) (P < 0.0001) (Fig. 1b) that revealed a 1.65-fold increase in β-catenin expression in malignant vs benign tumors. Regarding the β-catenin expression pattern in malignant and benign bone tumor subtypes, our data showed a considerable increase in the β-catenin mRNA expression level in osteosarcoma (5.581 ± 0.291), Ewing's Sarcoma (4.695 ± 0.2426), and chondrosarcoma (3.728 ± 0.2684) compared to their matched normal margins (P < 0.0001). The β-catenin expression level was prominent in osteosarcoma compared to Ewing's Sarcoma (P = 0.0236) and chondrosarcoma (P < 0.001) (Fig. 1c). The significant increase in the β-catenin mRNA expression level in osteochondroma (2.192 ± 0.1727), Giant Cell Tumor (3.650 ± 0.333), and exostosis (2.627 ± 0.1810) compared to their matched normal margin was observed (P < 0.0001) (Fig. 1d). Amongst the benign tumor subtypes, Giant Cell Tumors expressed a higher level of β-catenin mRNA compared to the osteochondroma (P = 0.0003) and exostosis (P = 0.0098) while the difference between exostosis and osteochondroma was not significant. Moreover, comparing each malignant subtype to each benign subtype showed a remarkable difference between the β –catenin mRNA expression in osteosarcoma with osteochondroma, Giant Cell Tumor and exostosis (P < 0.0001) groups. while; chondrosarcoma tumors expressed a significantly higher level of β-catenin gene compared to the osteochondroma (P < 0.05) and exostosis (P < 0.001) but no remarkable difference was observed regarding the chondrosarcoma and Giant Cell Tumor group. The β –catenin mRNA expression in Ewing's Sarcoma was significantly higher compared to osteochondroma (P < 0.0001). Giant Cell Tumor (P < 0.05) and exostosis (P < 0.0001) (Fig. 1e) groups. As illustrated in Fig. 2, the β-catenin mRNA level was 6.026 ± 0.3179 and 5.147 ± 0.2954 in tumor tissues of osteosarcoma and Ewing's Sarcoma of patients who received chemotherapy regimen, respectively. Patients who received chemotherapy showed a 1.27 and 1.26-fold increase in the β-catenin mRNA expression level compared to the patients with no history of chemotherapy in osteosarcoma and Ewing's Sarcoma groups, respectively (Fig. 2a). Notably, the level of cell differentiation compared to healthy bone cells is taken as a criterion of determining tumor grade, therefore, high-grade tumors are moderate or poorly differentiated compared to healthy bone cells. In comparing the expression level of β-catenin in high- and low-grade tumors, it was revealed that high-grade osteosarcoma (6.124 ± 0.2933, P = 0.013), Ewing's Sarcoma (4.649 ± 0.4111, P = 0.0046) and chondrosarcoma (5.246 ± 0.2937, P = 0.0057) tumors expressed higher mRNA level of β-catenin compared to the low-grade tumors in each group (Fig. 2b). An increase in the β-catenin mRNA expression level was detected in recurrent osteosarcoma (7.073 ± 0.3697, P = 0.0004) and recurrent Ewing's Sarcoma (5.847 ± 0.3671, P = 0.0020) tumors compared to their non-recurrent tumors that illustrate the approximate 1.43- and 1.36-fold increase in each group (Fig. 2c). Also, our data revealed that osteosarcoma (6.158 ± 0.4598) and Ewing's Sarcoma (5.410 ± 0.4165) tumors with poor response to chemotherapy based on Hovus grading expressed higher level of β-catenin mRNA level compared to the tumors with good response to chemotherapy; however, the observed differences were not statistically significant (Fig. 2d). Additionally, a significant increase in the β-catenin mRNA expression level was detected in metastatic tumors of osteosarcoma (6.636 ± 0.380, P = 0.0055), chondrosarcoma (4.681 ± 0.458, P = 0.0069), and Ewing’s Sarcoma (5.600 ± 0.301, P = 0.0015) which showed 1.30, 1.45- and a 1.35-fold increase compared to non-metastatic tumors in each group, respectively (Fig. 2e).

### The β-catenin expression level in peripheral blood of patients with different types of primary bone tumors

**Figure 3:**
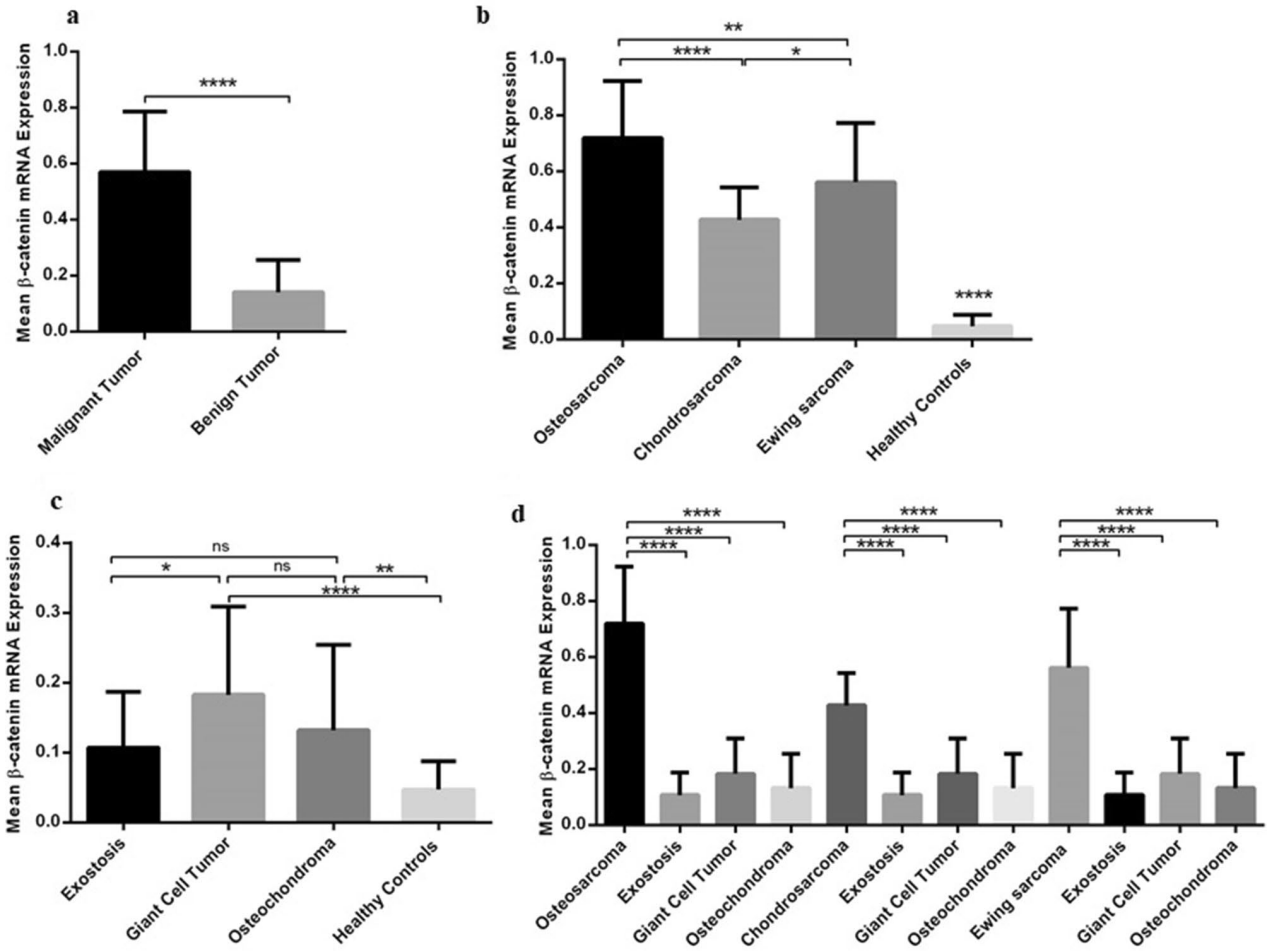
The β-catenin expression in peripheral blood of patients with bone tumors and healthy subjects. The β-catenin mRNA expression level was increased in PBMC of patients with different bone tumors. The expression level of β-catenin was increased in PBMC of patients with malignant bone tumors compared to PBMC of patients with benign bone tumors (**a**). The β-catenin gene expression level was enhanced in PBMC of patients with osteosarcoma, chondrosarcoma, and Ewing's Sarcoma (**b**), and in osteochondroma, Giant Cell Tumor, and exostosis compared to their matched normal controls (**c**). The β-catenin mRNA expression level in PBMC was increased in osteosarcoma, chondrosarcoma and Ewing's Sarcoma tumors compared to benign tumor subtypes subsequently (**e**). The t-student test was applied to evaluate the β-catenin gene expression level in the PBMC of patients with malignant bone tumors compared to benign bone tumors and comparison between their subtypes (**a**, **d**). To analyze the β-catenin expression level in PBMCs of patients with malignant bone tumors and healthy controls as well as patients with benign bone tumors and healthy controls, the one-way analysis of variance (ANOVA) was used (**c**, **d**). The comparative CT (2^−ΔCt^) method was applied for gene expression analysis. The statistical differences between groups are shown as asterisk (*P < 0.05, **P < 0.01, ****P < 0.0001).

**Figure 4:**
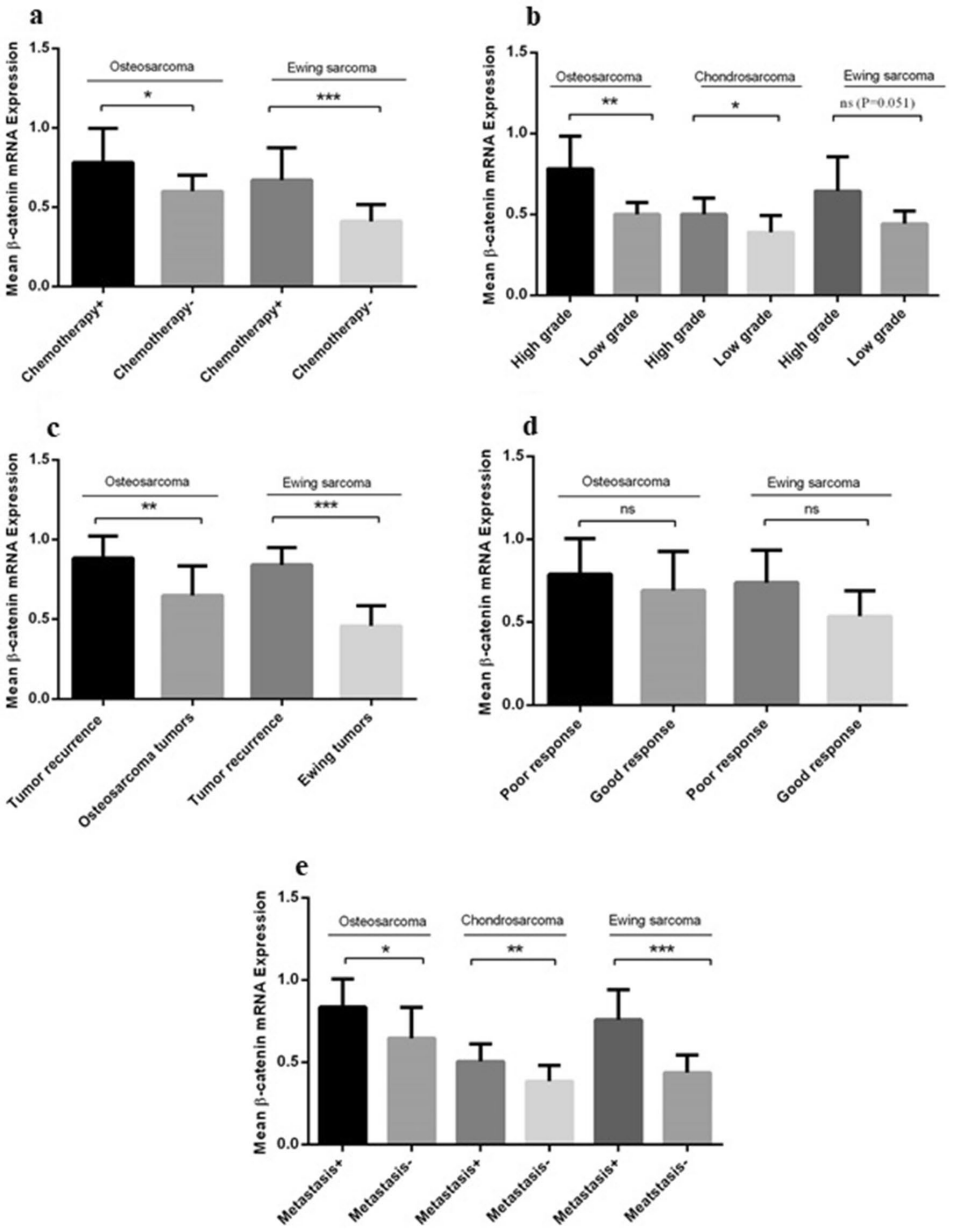
The circulating level of β-catenin expression in patients with different malignant bone tumors. The mRNA expression level of β-catenin in PBMCs of patients with different types of malignant bone tumors was assessed. The increase in the mRNA expression level of β-catenin was detected in PBMCs of chemotherapy-received patients with osteosarcoma and Ewing's Sarcoma tumors (**a**), PBMC of patients with high-grade osteosarcoma and chondrosarcoma tumors (**b**), PBMC of patients with recurrent osteosarcoma and Ewing's Sarcoma tumors (**c**). The β-catenin mRNA expression in PBMC of patients with poor response to chemotherapy of osteosarcoma and Ewing's Sarcoma tumors was not significant (**d**). The β-catenin expression level in PBMC of patients with metastatic osteosarcoma, Ewing's Sarcoma and chondrosarcoma tumors was increased compared to the non-metastatic groups (**e**). The t-student test was used to compare the difference in β-catenin expression in PBMC of patients with different malignant bone tumors and their subgroups. The comparative CT (2^−ΔCt^) method was applied for gene expression analysis. The statistical differences between groups are shown as asterisk (*P < 0.05, **P < 0.01, ***P < 0.001).

The expression level of β-catenin was assessed in PBMCs of the patients with different types of bone tumors due to the feasibility of liquid biopsy efficiency for early diagnosis. Based on our data, a significant increase in the mRNA expression level of β-catenin was detected in patients with malignant tumors (0.5695 ± 0.02443) compared to patients with benign tumors (0.1408 ± 0.01348) indicating a considerable fourfold increase (P < 0.0001) (Fig. 3a). Comparing the β-catenin mRNA expression level in patients with different types of malignant bone tumors showed an increase in the β-catenin mRNA expression level in osteosarcoma (0.7200 ± 0.0429), chondrosarcoma (0.4269 ± 0.0429) and Ewing's Sarcoma (0.5615 ± 0.04116) compared to the β-catenin mRNA level in PBMC of healthy subjects (P < 0.0001) (Fig. 3b). A higher level of β-catenin mRNA expression was observed in patients with osteosarcoma compared to chondrosarcoma (P < 0.0001) and Ewing's Sarcoma (P = 0.003) also in patients with Ewing's Sarcoma compared to chondrosarcoma (P = 0.042). The PBMC of benign bone tumors showed a significant statistical difference in β-catenin mRNA expression level between osteochondroma (0.1321 ± 0.02603, P = 0.004) and Giant Cell Tumor (0.1829 ± 0.02603, P = 0.0001) compared to the PBMC of healthy subjects which indicating 3.25 and 4.5-fold increase, respectively (Fig. 3c). The PBMCs of the patients with Giant Cell Tumor showed an elevated level of β-catenin mRNA expression level compared to patients with exostosis (P = 0.043) while the difference in the β-catenin expression between exostosis and osteochondroma also osteochondroma and Giant Cell Tumor group were not remarkable (Fig. 3c). Comparing the β-catenin mRNA expression in PBMCs of the patients with osteosarcoma, chondrosarcoma and Ewing's Sarcoma to each benign tumor subgroup showed remarkable differences for each tumor type (P < 0.0001) (Fig. 3d). The β-catenin mRNA expression in patients with osteosarcoma and Ewing's Sarcoma who received chemotherapy was 0.7829 ± 0.05227 (P = 0.0258) and 0.6713 ± 0.05229 (0.0007) compared to the patients without any history of chemotherapy, respectively (Fig. 4a). Also, the β-catenin expression in patients with high-grade osteosarcoma, chondrosarcoma and Ewing's Sarcoma was 0.7822 ± 0.04736 (P = 0.0062), 0.5000 ± 0.03221 (P = 0.0213) and 0.6450 ± 0.052(P = 0.0514), compared to its expression in patients with low-grade tumors, respectively; that illustrates the approximate 1.56- and 1.28-fold increase in the mRNA expression level of β-catenin in osteosarcoma and chondrosarcoma group (Fig. 4b). Comparing the level of β-catenin mRNA expression in patients with recurrent tumors, it was revealed that the expression was increased in patients with recurrent osteosarcoma (0.8844 ± 0.04562, P = 0.0025) and Ewing's Sarcoma (0.8414 ± 0.04108, P ≤ 0.0001) tumors comparing to non-recurrent tumors in each group with the 1.37- and 1.86-fold increase (Fig. 4c). Notably, the mRNA level of β-catenin in PBMC of the patients with poor response osteosarcoma (0.7892 ± 0.06219, P = 0.3702), and Ewing’s Sarcoma (0.7390 ± 0.0616, P = 0.0643) showed no significant difference compared to the tumors with good response in each group (Fig. 4d). However, the mRNA level of β-catenin in PBMC of the patients with metastatic osteosarcoma (0.8370 ± 0.05420, P = 0.0162), chondrosarcoma (0.5056 ± 0.0354, P = 0.0082) and Ewing’s Sarcoma (0.7600 ± 0.05804, P < 0.0001) showed a significant increase in the mRNA expression level of β-catenin compared to non-metastatic groups with the approximate1.29- and 1.31, 1.76-fold increase (Fig. 4e).

### The expression of the protein of β-catenin in bone tumor tissues

**Figure 5:**
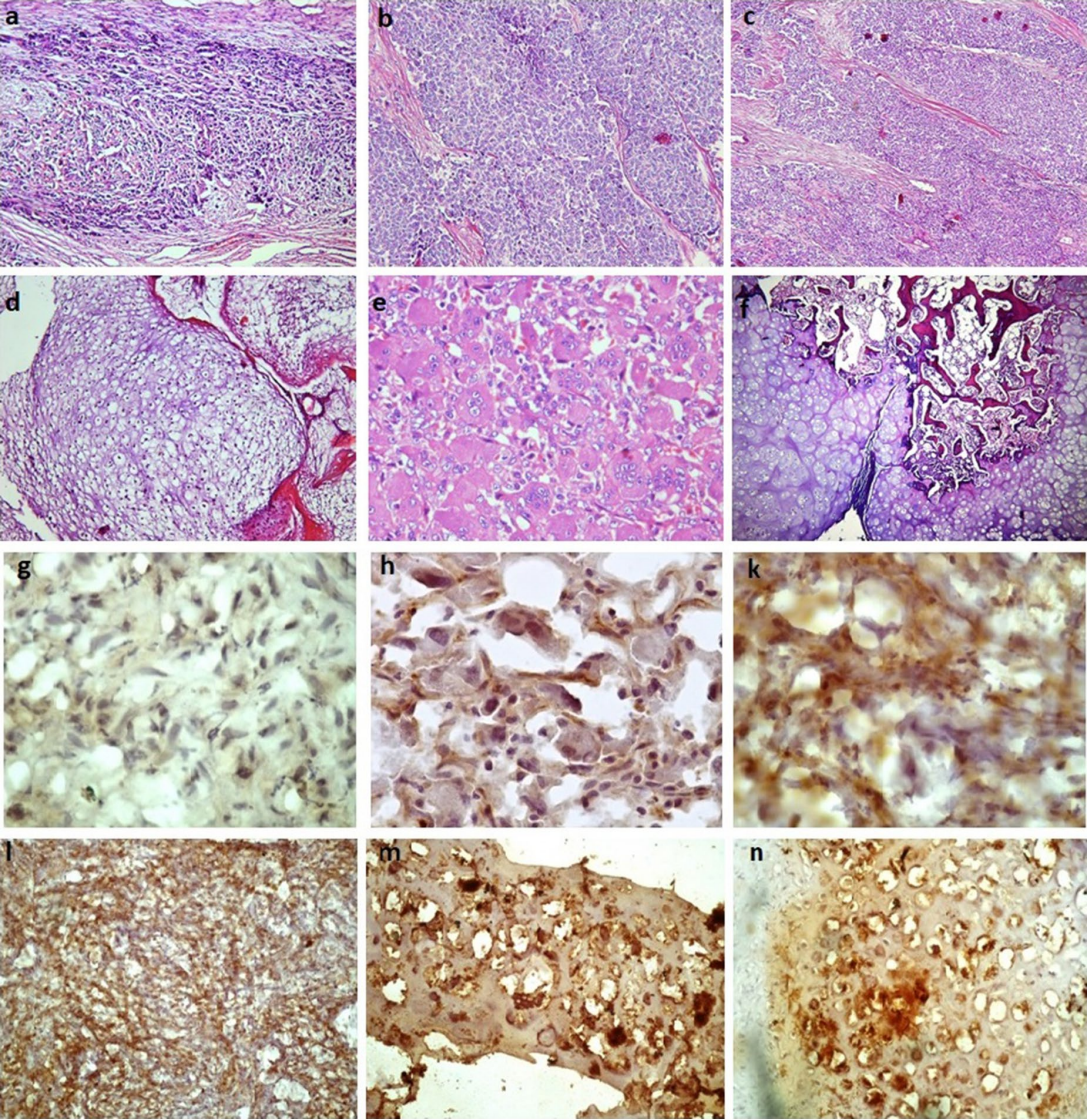
Primary bone tumor histology and immunohistochemistry staining of β-catenin protein. Histopathology with Hematoxylin and eosin (H&E) staining (**a**–**f**) and immunohistochemistry of β-catenin (**g**–**n**) in different bone tumor tissues were evaluated. The H&E staining of the osteosarcoma tumor tissue (**a**), Ewing sarcoma (**b**, **c**), chondrosarcoma (**d**), GCT (**e**) and osteochondroma (**f**) is illustrated. The negative immunoreactivity of β-catenin is shown in (**g**); while (**h**) represents GCT tumor tissue with the weak intensity of β-catenin staining. (**k**) and (**l**) represent GCT tumor tissues with moderate intensity of β-catenin staining, (**m**) represents chondrosarcoma and (**n**) represents osteosarcoma tumor tissue with strong intensity of β-catenin staining. Nuclear localization of β-catenin is shown in osteosarcoma tissue (**n**), cytoplasmic expression of β-catenin is shown in GCT tumor **(k**); while chondrosarcoma tumor tissue represents both nuclear and cytoplasmic expression of β-catenin (**m**). The scale of magnification: (**a**–**f**) 40; (**g**–**n**) 200.

β-Catenin, as an important mediator of the canonical Wnt signaling pathway, plays a decisive role in cell adhesion, movement and proliferation. Cytoplasmic accumulation and nuclear translocation of β-catenin indicates that the Wnt canonical pathway is activated^15^. Based on shreds of evidence, cytoplasmic and/or nuclear localization of β-catenin is associated with tumor transformation, malignancy and poor prognosis in different types of tumors^16,17^. Notably, it is well-established that phosphorylation of β-catenin at Ser33 and Ser37 and Thr47 by glycogen synthase kinase (GSK) leads to the proteosomal degradation of β-catenin and the non-phosphorylated β-catenin accounts as the active protein that mediates target gene transcription^18^. To determine whether the increased β-catenin mRNA expression level in bone tumor tissues is accompanied with its elevated protein level, the β-catenin protein level was assessed using immunohistochemistry in several types of malignant and benign bone tumors. The un-phosphorylated form of β-catenin that is known as the active form of this protein^19^ was assessed in the current study. The protein level of β-catenin was evaluated and scored by the pathologist and data are summarized in Table 3. Based on our data, 16.69% of malignant bone tumors revealed no β-catenin expression; while, 26.92%, 32.05% and, 24.35% of malignant tumors revealed the weak, moderate and strong intensity of β-catenin protein, respectively. The β-catenin protein expression showed poor and moderate intensity in 44.44% and 6.9% of benign tumors, respectively; while 48.61 of benign tumors showed negative expression of β-catenin protein. The difference in β-catenin protein expression between malignant and benign bone tumors was statistically significant (P < 0.0001). Accordingly, 11.03%, 23.07% and 15.38% of osteosarcoma, Ewing's Sarcoma and chondrosarcoma tumors revealed negative β-catenin expression, respectively; while β-catenin weak intensity was detected in 19.23%, 30.76% and 45.83%, β-catenin moderate intensity was detected in 30.76%, 34.61% and 30.76%, and β-catenin strong intensity was detected in 38.46%, 11.53% and 23.07% of osteosarcoma, Ewing's Sarcoma and chondrosarcoma tumors, respectively. Although, it seems that osteosarcoma tumors showed a higher intensity of β-catenin protein expression, the difference between malignant bone tumor types regarding the expression of β-catenin protein was not significant statistically (P = 0.433). Regarding malignant bone tumors, in patients who received chemotherapy, 3.125%, 12.5%, 46.87% and 37.5% of tumors showed negative, poor, moderate and strong β-catenin protein expression, respectively; while, 30.43%, 36.95%, 23.91% and 8.69% of tumors with no history of chemotherapy revealed negative, poor, moderate and strong β-catenin protein expression, respectively. Data indicated a significant difference between chemotherapy-positive and negative tumors regarding β-catenin protein expression (P < 0.0001). In metastatic malignant tumors, 3.44%, 37.93% and 58.62% revealed the negative, moderate and strong intensity of β-catenin protein, respectively; while 24.48%, 42.85%, 28.57% and 4.08% of non-metastatic malignant bone tumors revealed negative, poor, moderate and strong β-catenin protein expression. As it is indicated none of the metastatic tumors showed weak β-catenin protein expression and a considerable difference in β-catenin protein expression between metastatic and non-metastatic bone tumors was detected (P < 0.0001). In addition, 26.66% and 73.33% of recurrent bone tumors showed moderate and strong β-catenin protein expression, respectively and none of these tumors showed negative and weak β-catenin protein expression. However, 26.47%, 38.23%, 32.45% and 5.88% of non-recurrent bone tumors revealed negative, poor, moderate and strong β-catenin protein expression, respectively. The difference between β-catenin protein expression in recurrent and non-recurrent bone tumors was statistically significant (P < 0.0001). Also, in high-grade malignant tumors, 2.27%, 9.09%, 45/45% and 43.8% of tumors showed negative, poor, moderate and strong β-catenin protein expression, respectively; while, 31.81%, 45.45%, 22.72% of malignant tumors with low grade showed negative, poor, and moderate β-catenin protein expression and none of these tumors showed strong intensity of β-catenin protein. Based on data, a considerable difference between high- and low-grade tumors regarding β-catenin protein expression was detected (P < 0.0001). Regarding β-catenin localization, both cytoplasmic and nuclear expression of β-catenin was detected in bone tumor tissues; while nuclear localization was detected in malignant tumors (Fig. 5n) predominantly and cytoplasmic localization was found in benign tumors (Fig. 5k). The histopathology of bone tumor tissues was evaluated using hematoxylin and eosin (H&E) staining (Fig. 5a–f). Moreover, the representative images of β-catenin immunohistochemistry staining in primary bone tumor tissues are illustrated in Fig. 5g–n.

### Association of β-catenin expression with the demographic features of the patients with diverse types of bone cancer

To better determine how the local and circulating mRNA expression level of β-catenin, the protein expression, and patient's demographic features are related, the correlation of β-catenin expression with the aforementioned factors was evaluated for each tumor type separately and the results are presented in Supplementary Tables S1–Tables S6. Regarding the osteosarcoma tumors, it was revealed that the β-catenin gene expression in the tumor was significantly correlated with its circulating expression (P = 0.019) and protein expression (P = 0.001) also the β-catenin expression in PBMCs was correlated with β-catenin protein expression in tumor (P = 0.004). Notably, the osteosarcoma tumor size was significantly correlated with β-catenin gene expression in the tumor tissues (P = 0.03); while no significant correlation was observed regarding the tumor size and β-catenin gene expression in PBMCs of the patients and also β-catenin protein expression in tumor tissues. The osteosarcoma tumor grade was positively correlated with β-catenin gene expression in osteosarcoma tumors (P = 0.013), in patients’ PBMCs PBMC (P = 0.007) and local protein expression (P = 0.001). Also, the level of tumor response to the chemotherapy was correlated with the β-catenin gene expression (P = 0.003) and β-catenin protein expression in tumor tissue (P = 0.001); while it showed no correlation with β-catenin gene expression in patient’s PBMC. Also, a metastatic feature of osteosarcoma tumors was statistically correlated with β-catenin gene expression in tumor tissue (P = 0.004), in patients’ PBMCs (P = 0.022), and local protein level (P < 0.0001). The β-catenin gene expression in the tumor (P < 0.0001), PBMCs (P = 0.007), and its local protein expression (P < 0.0001) were correlated significantly with osteosarcoma recurrent tumors. However, no significant correlation was observed between the β-catenin gene and protein expression and osteosarcoma patient’s age (Supplementary Table S1). Regarding the chondrosarcoma tumors (Supplementary Table S2), β-catenin gene expression in chondrosarcoma tumor tissues showed no specific correlation with β-catenin circulating level and protein expression in tumor tissues. While the β-catenin gene expression in patients’ PBMC was positively correlated with β-catenin protein expression in chondrosarcoma tumor tissues (P = 0.006). The chondrosarcoma tumor size was significantly correlated with the β-catenin gene expression in the tumor tissue (P = 0.032), patients’ PBMCs (P = 0.015), and tumor tissue protein expression (P ≤ 0.0001). Notably, the chondrosarcoma tumor grade showed a significant correlation with the β-catenin gene expression level in tumor tissue (P = 0.005) in patients’ PBMCs (P = 0.021) and its protein expression in tumor tissue (P < 0.0001). The same pattern was observed regarding the metastatic feature of chondrosarcoma tumors and β-catenin gene expression in tumor tissue (P = 0.011), in PBMCs (P = 0.013), and its protein expression in tumor tissue (P ≤ 0.0001). However, the correlation of the chondrosarcoma patients’ age was only detected with β-catenin gene expression in patients’ PBMCs (P = 0.003). Regarding the Ewing’s Sarcoma tumors, the correlation analysis showed that (supplementary table S3), the β-catenin gene expression in tumor tissues was correlated significantly with β-catenin expression in patients’ PBMCs (P < 0.0001) and protein expression in tumor tissues (P < 0.0001), also the β-catenin protein expression in tumor tissue was correlated with β-catenin circulating level (P < 0.0001). The Ewing’s Sarcoma tumor size was correlated with the level of β-catenin gene expression in tumor tissue (P < 0.0001), in patients’ PBMCs (P = 0.018), and β-catenin protein expression in tumor tissue (P = 0.016). However, the Ewing’s Sarcoma tumor grade was only correlated significantly with the β-catenin gene expression (P = 0.002) and protein expression (P = 0.006) in tumor tissues; while showed no significant correlation with β-catenin gene expression in patients’ PBMCs. Also, it was revealed that tumor grade of necrosis following chemotherapy was not significantly correlated with the β-catenin gene/protein expression in tumor tissues and negatively correlated with its expression in patients’ PBMCs (P = 0.010). However, the metastatic status of Ewing’s Sarcoma tumors illustrated considerable correlation with the β-catenin gene expression in tumor tissue (P = 0.001), in patients’ PBMCs (P ≤ 0.0001), and β-catenin protein expression in tumor tissues (P = 0.001). In accordance, the recurrent Ewing’s Sarcoma tumors showed a strong correlation with the β-catenin gene expression in tumor tissue (P = 0.002), patients’ PBMCs (P ≤ 0.0001), and β-catenin local protein expression in tumor tissues (P ≤ 0.0001). Ultimately, no specific correlation was detected between the patients’ age and β-catenin gene and protein expression in Ewing’s Sarcoma. Regarding osteochondroma tumors (Supplementary Table S4), the β-catenin gene expression in osteochondroma tumors was not significantly correlated with its expression level in patients’ PBMCs and protein expression in tumor tissues. Also, no correlation was found regarding the patient's age and β-catenin gene and protein expression in tumor tissue and patients’ PBMCs in osteochondroma patients. The osteochondroma tumor size was significantly correlated with the β-catenin protein expression in tumor tissues (P = 0.029); while showed no significant correlation with β-catenin gene expression in tumor tissues and patients’ PBMCs. Regarding Giant Cell tumors (Supplementary Table S5), the β-catenin gene expression in tumor tissue was correlated with its protein expression in tumor tissues (P = 0.009); while no correlation was detected regarding the β-catenin gene expression in tumor tissue and patients’ PBMCs also β-catenin protein expression in tumor tissue and its gene expression in patients’ PBMCs. Giant Cell Tumor size was significantly correlated with the β-catenin gene expression in tumor tissue (P = 0.015), patients’ PBMCs (P = 0.03) and β-catenin protein expression in tumor tissue (P = 0.002). Also, no significant correlation was detected regarding the patients’ age and β-catenin gene and protein expression in patients with Giant Cell tumor. In exostosis tumors (Supplementary Table S6), no correlation was found regarding the β-catenin gene expression in tumor tissue in patients’ PBMC sand β-catenin protein expression in tumor tissues. Also, no correlation was observed between patients age and the local and circulating level of β-catenin gene and protein in patients with exostosis. Also, based on our data, exostosis tumor size was not significantly correlated with β-catenin gene and protein expression in tumor tissue and patients’ PBMCs. Additionally, according to the obtained regression model, increasing the expression of β-catenin protein along with other variables increases the risk of metastasis of bone tumors (for each unit of increased protein expression, the probability of metastasis increases 5.2 times). Expression of β-catenin gene in tumor tissue (OR = 2.3, 95% CI 1.53–3.58, P-value < 0.0005) as well as expression of β-catenin in PBMC (OR = 3.23, 95% CI 1.79–5.82, P-value < 0.0005) alone can be effective in predicting metastasis; however, in the obtained regression model, OR was not significant. Also, in predicting the growth rate of bone tumors (grade), the gene expression of β-catenin and its protein expression in tumor tissue can be effective in tumor growth rate. Thus, increasing the expression of β-catenin gene as well as protein in bone tumor tissue leads to an increase in the likelihood of incidence of higher grade bone tumors (For every unit increase in β-catenin gene expression in bone tumor tissue, the probability of higher grades tumor incidence is 2.3 times higher, and for every increase in protein expression of β-catenin in bone tumor tissue, the probability of higher grades tumor incidence increases 6.7 times). The expression of β-catenin gene in PBMC (OR = 7.3, 95% CI 2.33–22.9, P-value < 0.005) alone can play a role in predicting the rate of disease progression (Grade) but is not significant in the regression model. Other variables in the regression model did not show a significant predictive value and therefore the results are not shown (Supplementary Table S7).

## Discussion

It is taken for granted that bone is avascular and fibrous tissue with the ability to change its cell profile and is a suitable substrate for the placement of circulating tumor cells. On the other hand, common and traditional therapeutic approaches for primary bone tumor treatments limit the efficacy and efficiency of the definitive treatment of this disease. Accordingly, genomic characterization of tumors besides unraveling intracellular pathways that are involved in tumor pathogenesis, might pave the way to introduce actionable and impressive molecular targets as new therapeutic goals. β-catenin as the main mediator of the canonical Wnt pathway, gained considerable attention due to its involvement in important aspects of cellular processes such as cell invasion, migration, proliferation, and differentiation. Also, the regulatory role of β-catenin in the epithelial-mesenchymal transition (EMT) process, as a critical step of tumor cell invasion, emphasizes its important role in cancer cell fate. Considering the fundamental and decisive role of β-catenin in tumor cell formation and tumor progression and the fact that β-catenin is a functional readout of the Wnt pathway that can be modulated and targeted, its local and circulating expression level was investigated in six types of primary bone tumors with different patient’s clinic pathological features, in the current study. Based on our data, a considerable increase in the mRNA and protein expression of β-catenin was observed in primary bone tumors compared to bone normal tissues. The increase in the β-catenin expression was apparent in osteosarcoma, Ewing's Sarcoma, and chondrosarcoma compared to the matched tumor margins, in which the higher expression of β-catenin was detected in osteosarcoma tumors compared to the other malignant bone tumors. In line with our evidence, it was shown that β-catenin expressed more in osteosarcoma compared to osteochondroma ^20^. Also, the lower expression of β-catenin in Ewing’s Sarcoma tumors was detected compared to osteosarcoma tumors indicating that the overall impact of Wnt mediators in Ewing’s Sarcoma is driving differentiation rather than proliferation ^13^. It was shown that up-regulation of β-catenin is a genetic feature that is associated with metastasis of tumor cells since the decrease in β-catenin expression and transcription level caused attenuation of the metastasis-associated phenotypes of breast cancer cells ^21^. Also, the association of β-catenin/Wnt pathway knock-down with increased apoptosis restoration is postulated in tumor cells^22^. Besides, it was proven that β-catenin is involved in the regulation of cancer stem cell marker genes that play a functional role in cell renewal ability and stemness features that all together suggest that β-catenin may critically act in fostering chemo resistance of tumor cells ^23,24^. It was shown that utilizing β-catenin transcription inhibitors can sensitize osteosarcoma cell lines to the chemotherapeutic agents revealing the impactful role of β-catenin in tumor response to the chemotherapy treatment ^25^. Notably, activation and/or reactivation of tumor cells after the remission period accounts for a challenging and perilous issue in cancer management which might be occurred in up to 65% of bone cancers ^26^. In accordance, to get insights into the correlation of β-catenin expression level with tumor metastasis, recurrent and chemo resistance, our data revealed that an increase in the expression level of β-catenin was associated with tumor metastasis, recurrent and poor response to chemotherapy in osteosarcoma, and Ewing's Sarcoma which is in line with previous evidence. In the current study, patients with chondrosarcoma tumors had no history of chemotherapy and none of them had recurrent tumors; while an increase in β-catenin expression level was also correlated with tumor metastasis in the chondrosarcoma group. Also, based on our data, the elevated expression of β-catenin protein (intensity level) along with other variables increases the risk of metastasis and tumor grade of bone tumors. Also, it was found that β-catenin suppression/ degradation inhibited cell proliferation and invasion as well as induced apoptosis in renal cell carcinoma ^27^ and lung cancer cell ^28^. Moreover, multiple lines of evidence indicate that activation of the β-catenin pathway promotes cell proliferation, invasion, and migration through different mechanisms such as Long noncoding RNAs regulation ^29^ and interaction with histone demethylase ^30^. In the current study, an increase in the β-catenin expression was associated with malignant tumor grade and size indicating the possible positive role of β-catenin in enhancing primary bone tumor proliferation and growth. In support of our data, it was delineated that the interaction of β-catenin with forkhead box protein M1 (FOXM1) mediates osteosarcoma cell growth and metastasis ^31^. To clarify whether the β-catenin expression pattern can be affected by tumor type; the most prevalent benign bone tumors such as Giant Cell Tumor, osteochondroma, and hereditary multiple exostosis were also included in this survey and our data revealed a significant increase in β-catenin expression level in benign bone tumors compared to normal bone tissues, also malignant tumors expressed a higher level of β-catenin significantly compared to benign bone tumors;. Based on our data, Giant Cell Tumors expressed a higher level of β-catenin compared to the other benign bone tumors and showed a significant association between tumor size and β-catenin expression level. The relevance of β-catenin function in bone hemostasis, metabolism, and turnover has been illustrated in previous studies. It was shown that Wnt canonical pathway is involved in the osteogenesis process specifically via sclerostin and cell surfaces proteins such as ZNRF3 and RNF43^32^. Also, activation of β-catenin/Wnt pathway caused elevation of osterix and osteoprotegerin expression level which promoted osteoblast differentiation and osteoclast suppression, respectively ^33^. Several findings support the fact that peripheral blood can be served as a possible circulating source of biomarkers and a less invasive diagnostic route that can reflect the immune system behavior and response in pathologic situations ^34^. Also, as a possible circulating source of cancer cells/cancer stem cells that have been released in the blood, perusing peripheral blood isolated cells beside tumor tissues might provide a better and more comprehensive picture of molecular targets and immune cells alterations in a pathological situation such as cancer ^35^. In accordance, the mRNA expression assessment of β-catenin in PBMCs of patients with malignant and benign bone tumors revealed an increase in the expression level of β-catenin in malignant compared to benign tumors. The same expression pattern of β-catenin was observed in tumor and PBMCs of each malignant tumor type compared to normal bone tissues, also patients with osteosarcoma and Giant Cell Tumors expressed a higher level of β-catenin compared to the rest of tumor types in their group, respectively. Based on our data, the circulating level of β-catenin in malignant tumors was correlated with tumor metastasis, recurrent, and grade. Also, based on the fact that the mRNA level might be influenced by different genetic and epigenetic processes that can cause loss of protein expression ^36^, in the current study, the local active form of β-catenin protein was also assessed in malignant and benign tumors. The overexpression of β-catenin protein was detected in malignant compared to benign bone tumors and the β-catenin expression was associated with tumor grade, metastasis, recurrent, and response to therapy. Therefore, the β-catenin gene and protein expression revealed consistency in different types of primary bone tumors also the simultaneous increase in the expression of β-catenin in tumor site and patient's PBMCs indicates the possible role of β-catenin as a diagnostic marker in bone cancer. The correlation of β-catenin expression with patients' age was only detected in PBMCs of patients with chondrosarcoma which can be explained by the fact that in the chondrosarcoma group, all of the participants were at the same age range (> 20 years). Taken together, assessing the simultaneous local and circulating expression pattern of β-catenin in diverse types of malignant and benign bone tumors at both gene level and protein expression underlines the possible relevance of β-catenin to primary bone cancer pathogenesis, however, is required further mechanistic studies and clinical studies with more patients. Also, survival analysis on patients with varying degrees of tumor severity and response to treatment in relation to β-catenin expression can also provide valuable information.

## Methods

### Patients and sample collection

The number of 150 bone tumor tissues, 150 tumor margins, and 150 peripheral drops of blood taken from the same patients with bone tumor as well as 32 healthy blood samples were enrolled in the current study with local ethical approval and informed consent. The project was ethically approved by the ethics committee of the Vice president of research of the Iran University of Medical Sciences with the ethics committee code: IR.IUMS.REC 1395.95-04-30-29364. Written informed/Informed consent was obtained from all participants before enrolling in the study and for those patients whose age was less than 18 years, consent was submitted from their legal guardian and after approval and signing of consent, the patient was included in the survey. Signed informed consent of participants will be provided upon request. Also, all procedures and methods were performed following the relevant guidelines and regulations with the ethical standards of the responsible committee on human experimentation in Iran University of Medical Sciences and in accordance with the Helsinki Declaration^37^. A pair of the tumor and marginal tissue samples were taken from all the patients who were subjected to surgery at Shafa Orthopedic Hospital and were kept immediately at − 80 for later assays. The tumor and normal bone tissue collection and processing were followed as in our previous study^38^. For evaluation of circulating factors, the 6 ml peripheral blood was taken from the patients as well as healthy controls and used for peripheral blood mononuclear cell (PBMC) separation. The patients and healthy subjects were matched as a matter of age and sex. In the current study three types of common malignant bone tumors including osteosarcoma as a deadly form of bone tumors, Ewing's Sarcoma as a small round cell tumor and chondrosarcoma as a malignant tumor consisting of chondrocytes were included^39^. Also, three types of benign bone tumors including Giant Cell Tumors which are also known as osteoclastomas**,** osteochondroma as benign single chondrogenic lesions and hereditary multiple exostosis as a genetic disorders consisting of multiple osteochondromas were included^40^. The clinic-pathological features of patients are summarized in Table 1. The variables are presented as the number of patients and percentages in each group of tumors separately and the analysis shown for each demographic feature in this table is the difference between tumors groups. In brief, the number of patients with prevalent types of malignant bone tumors including osteosarcoma, Ewing's Sarcoma, and chondrosarcoma was equal in the study. As it is shown in Table 1, 46.2% and 53.8% of patients with osteosarcoma and Ewing's Sarcoma were under 20 years of age, respectively; while all of the patients with chondrosarcoma were above 20 years of age. Also, 50%, 61.5%, and 53.8% of the enrolled patients with osteosarcoma, Ewing's Sarcoma, and chondrosarcoma were male, respectively. The size of tumors in 72.7%, 57%, and 49.9% of patients with osteosarcoma, Ewing's Sarcoma, and chondrosarcoma was over 8 cm, respectively. Additionally, 78.3%, 76%, and 45.4% of the patients had high-grade tumors in osteosarcoma, Ewing's Sarcoma, and chondrosarcoma, subsequently. In the current study, 38.5% of osteosarcoma, 38.5% of Ewing's Sarcoma, and 34.6% of chondrosarcoma tumors were metastatic. Also, 65.4% and 57.7% of patients with osteosarcoma and Ewing's Sarcoma tumors received chemotherapy treatment before the surgery, respectively; while none of the patients with chondrosarcoma received chemotherapy regimens. Notably, the standard combination of doxorubicin, cisplatin, and methotrexate was applied for osteosarcoma patients while the combination of vincristine, cyclophosphamide, and doxorubicin was used for Ewing's Sarcoma patients, as chemotherapy approaches. To follow up the efficiency of the chemotherapy regimen, the histological response to the chemotherapy treatment was determined based on the tumor necrosis over 95% as a cutoff value using the Huvos system grading. In accordance, little or partial response to the chemotherapy causes a low rate of tumor necrosis and shows as grade 1/2 while a good response to the chemotherapy results in tumor necrosis over 95% and shows as grade 3/4^41^. In the current survey, 63.2% of patients with osteosarcoma and 66.7% of patients with Ewing's Sarcoma showed poor response to the chemotherapy treatments. Also, 33.4% of patients with osteosarcoma and 26.9% of Ewing's Sarcoma patients had recurrent tumors while none of the patients with chondrosarcoma had recurrent tumors. The difference between chondrosarcoma and the other two malignant tumor groups as a matter of age (P < 0.0001) tumor grade (P = 0.035), and chemotherapy received/not received (P < 0.0001) was statistically significant. Also, the difference between osteosarcoma and chondrosarcoma as a matter of tumor size (0.029) was statistically significant, while the rest of the features showed no specific difference between malignant bone tumor groups. Additionally, Table 2 illustrates the clinical features of patients with benign bone tumors. Briefly, 25%, 29.2%, and 45.8% of patients with osteochondroma, Giant Cell Tumor, and exostosis were under 20 years of age. In patients with benign tumors, 66.7%, 62.5%, and 58.4% of osteochondroma, Giant Cell Tumor, and exostosis were male, respectively. It was indicated that the tumor size in 84.2%, 89.5%, and 90.47% of patients with osteochondroma, Giant cell Tumor, and exostosis was below 8 cm. As a matter of age, gender, and tumor size, no statistical difference was observed between osteochondroma, Giant cell Tumor, and exostosis groups.

**Table 1:** The clinic- pathological features of patients with malignant bone tumors.

Demographic features	Groups	Osteosarcoma (N = 26)	Ewing’s sarcoma (N = 26)	Chondrosarcoma (N = 26)	P-value
Age^b^	≤ 20	12 (46.2%)	14 (53.8%)	0	< 0.0001
	> 20	14 (53.8%)	12 (46.2%)	26 (100%)	
Gender	Male	13 (50%)	16 (61.5%)	14 (53.8%)	0.69
	Female	13 (50%)	10 (38.4%)	12 (46.2%)	
Tumor size^a,c^ (cm)	≤ 8	6 (27.3%)	9 (42.8%)	13 (59%)	0.029*
	> 8	16 (72.7%)	12 (57%)	9 (40.9%)	
Tumor grade^a,b^	Low (grade I/II)	5 (21.7%)	5 (24%)	12 (54.5%)	0.035*
	High (grade III)	18 (78.3%)	16 (76%)	10 (45.4%)	
Chemotherapy^b^	Positive	17 (65.4%)	15 (57.7%)	0	< 0.0001
	Negative	9 (34.6%)	11 (42.3%)	26 (100%)	
Metastasis	Yes	10 (38.5%)	10 (38.5%)	9 (34.6%)	0.94
	No	16 (61.5%)	16 (61.5%)	17 (65.3%)	
Huvos grade^a,d^	Grade 1/2	12 (63.2%)	10 (66.7%)	0	0.83
	Grade 3/4	7 (36.8%)	5 (33.3%)	0	
Tumor recurrence^d^	Yes	8 (30.7%)	7 (26.9%)	0	0.76
	No	18 (69.2%)	19 (73.07%)	0	

^a^Number of patients with available information

^b^The observed difference is between chondrosarcoma and the other two groups.

^c^The observed difference is between Osteosarcoma and Chondrosarcoma.

^d^The observed difference is between Osteosarcoma and Ewing sarcoma.

**Table 2:** The clinic- pathological features of patients with benign bone tumors.

Demographic features	Groups	Osteochondroma (N = 24)	Giant cell tumor (N = 24)	Exostosis (N = 24)	P-value
Age	≤ 20	6 (25%)	7 (29.2%)	11 (45.8%)	0.17
	> 20	18 (75%)	17 (70.8%)	13 (54.2%)	
Gender	Male	16 (66.7%)	15 (62.5%)	14 (58.4%)	0.83
	Female	8 (33.3%)	9 (37.5%)	10 (41.6%)	
Tumor size^a^ (cm)	≤ 8	16 (84.2%)	17 (89.5%)	19 (90.47%)	0.571
	> 8	3 (15.7%)	2 (10.5%)	2 (9.5%)	

^a^Number of patients with available information.

### Peripheral blood mononuclear cell (PBMC) separation

To separate peripheral blood mononuclear cells (PBMCs) from the collected blood of patients and healthy controls, Ficoll-Hypaque (Sigma Chemical Co, St Louis, MO, USA) was utilized with subsequent density gradient centrifugation. The separated cells were washed with phosphate-buffered saline (PBS) and counted by hemocytometer. An equal number of PBMCs was applied for further assessment.

### RNA extraction, cDNA synthesis, and real-time PCR

Evaluation of β-catenin gene expression level was assessed using quantitative real-time PCR. Accordingly, the tumor, adjacent noncancerous tissues, and PBMCs were subjected to the RNA extraction via Trizol (Invitrogen, Grand Island, USA) based on the manufacturer’s instructions. Briefly, 700 µl of Trizol lysis reagent was used to lysis and homogenize bone tissues, after a sufficient incubation period, chloroform was added to each tissue homogenate for subsequent phase separation. The aqueous phase was collected and mixed with isopropanol. Following incubation and centrifugation, the remaining pellet containing RNA was washed with 75% ethanol, air-dried, and dissolved in RNase-free water. The Nanodrop spectrophotometer (Nanodrop Technologies) was applied for evaluating the quantity and quality of the RNA extracted from each sample. The cDNA synthesis was implemented using the PrimeScript First Strand cDNA Synthesis Kit (Takara, Japan) fro1 µg of the extracted RNA based on the manufacturer’s instructions. The SYBR Premix Ex Taq II (Takara, Japan) was used for assessment of β-catenin gene expression which was performed in Applied Biosystems Step One Plus, Real-time system (Applied Biosystems, USA). For detection of β-catenin gene level, the specific primers were designed and the beta-actin expression level was considered as a housekeeping gene. The sequence and characters of primers were as: Catenin forward primer: 5′-TCACTCCTCCTAATGGCTTG-3′, Catenin reverse primer: 5′-GTTGCTGCCAGTGACTAACA-3′ (Tm = 58), beta-actin forward primer: 5′-GAT CTC CTT CTG CAT CCT GT-3′, beta-actin reverse primer: 5′-TGG GCA TCC ACG AAA CTA C-3′ (Tm = 57). The primer’s specificity was determined by melting curve analysis for each amplified product. The running PCR program was as 1 cycle at 95 °C for 5 min following 40 cycles at 95 °C for 5 s, 55 °C for 20 s and 60 °C for 35 s. The PCR products were measured and approved their product length by electrophoresis. The comparative CT (2^−ΔCt^) method was applied for the analysis of gene expression.

### Tissue histopathology and Immunohistochemically staining of β-catenin

The Hematoxylin and eosin (H&E) histological staining was applied to identify bone cancerous and non-cancerous tissues and determine tissue composition, affinity and interaction to the dye^42^. To this aim, tissue sections were dehydrated for 5 min with alcohol and after washing with water, they were stained for 10 min with Harris’s hematoxylin stain. Tissues were washed with water and were subjected to differentiation in acid alcohol and incubated in lithium carbonate for 5 min. The tissues were stained in eosin for 15 s then dehydrated with graded alcohol and xylene and mounted. The expression of non-phosphor-β-catenin which is the stable and functional form of this protein was evaluated in tumor and normal bone tissues via immunohistochemistry. In summary, bone tissues were fixed and incubated in 4% paraformaldehyde and 20% sucrose, respectively. The Optimal Cutting Temperature (OCT) embedding medium was applied for preparing the frozen tissue blocks following incubation at − 27 °C. Tissue sections were prepared using cryotome and tissue slides were exposed to the washing buffer, H_2_O_2_ solution, Triton5%, and blocking buffer before staining by β-catenin primary antibody. The non-phosphor- β-catenin rabbit monoclonal antibody (Cell Signaling, the Netherlands, category number: 8814) was used in a dilution of 1:700, and tissue slides were incubated at a required time. The anti-rabbit IgG HRP-conjugated secondary antibody (Cell Signaling, the Netherlands and 3, 3′-diaminobenzidine (DAB, Dako) were used to visualize the primary antibody binding. For final dehydration and subsequent mounting, ethanol and xylolin were applied. The stained tissues were observed and scored by pathologists who were blinded to the data. The staining intensity method was applied to determine the immunoreactivity of β-catenin antibody and it was indicated as the absence of immunoreactivity; weak intensity; moderate intensity; and strong intensity in five different hot spot areas.

### Statistical analysis

For gene expression analysis, the comparative CT (2^−ΔCt^) method was used wherein ΔCt indicates the subtract of β-catenin Ct from the Ct of the endogenous gene (β-Actin). To determine whether the results of β-catenin gene expression are normally distributed in patients (tumor, margin and PBMC) also in the PBMC of the healthy group, the Kolmogorov–Smirnov analysis was applied before analyzing the difference in expression of β-catenin between different groups. Due to the normal distribution, the difference in the expression level of β-catenin was analyzed using t-student test between tumor and margin, malignant and benign tumors, malignant and benign tumor subgroups and their matched normal margins also malignant tumors with different tumor features (chemotherapy history, response to therapy, grade, metastasis status and recurrence) (Results are illustrated in Figs. 1 and 2). The same analysis procedure (t-student test) was used to compare the difference in β-catenin expression in PBMC of patients and their subgroups (Results are illustrated in Figs. 3a and 4). The one-way analysis of variance (ANOVA) which is used for comparisons with three or more groups was applied to analyze the β-catenin expression level in PBMCs of patients with malignant bone tumors and healthy controls as well as patients with benign bone tumors and healthy controls (Results are illustrated in Fig. 3b,c).The enumeration data are presented as percentages in the relevant tables and to clarify whether the difference between enumeration data in different groups are statistically significant, the chi-square test was used (Table 3). To analyze the difference between the clinic- pathological features of patients with malignant bone tumors and benign bone tumors Kruskal–Wallis H test was used to evaluate the differences between groups in terms of qualitative characteristics such as tumor recurrence, metastasis and chemotherapy and response to treatment and ANOVA statistical test was used to evaluate the differences between groups in terms of quantitative characteristics such as age and tumor size (Tables 1 and 2).The correlation of β-catenin expression level with patients' clinic pathological characteristics and tissue gene expression, PBMC gene expression, and protein level in different types of malignant and benign bone tumors was assessed using Spearman correlation coefficient test (Results are illustrated in Supplementary Tables S1–Tables S6). The logistic regression analysis was applied to determine the value of the variables in predicting β-catenin expression level in tumors that the results are shown in Supplementary Table S7. The Graph Pad Prism version 6 (Graph Pad Software, San Diego California) and Statistical Package for Social Science (SPSS v.16) were used for the calculation of all statistics. P values < 0.05 (two-sided) were considered statistically significant.

**Table 3:** The immunohistochemistry results of β-catenin protein evaluation in patients with different types of bone cancer.

Groups	Expression pattern^a^									P value
	Total number	Absence of immunoreactivity (negative)		Weak intensity		Medium intensity		Strong intensity		
		N	%	N	%	N	%	N	%	
Malignant tumors	78	13	16.69%	21	26.92%	25	32.05%	19	24.35%	< 0.0001
Benign tumors	72	35	48.61%	32	44.44%	5	6.9%	0	0	
Chemotherapy-received	32	1	3.125%	4	12.5%	15	46.87%	12	37.5%	< 0.0001
Chemotherapy-not received	46	14	30.43%	17	36.95%	11	23.91%	4	8.69%	
Metastatic tumors	29	1	3.44%	0	0	11	37.93%	17	58.62%	< 0.0001
Non-metastatic tumors	49	12	24.48%	21	42.85%	14	28.57%	2	4.08%	
Recurrent tumors	15	0	0	0	0	4	26.66%	11	73.33%	< 0.0001
Non-recurrent tumors	34	9	26.47%	13	38.23%	11	32.45%	2	5.88%	
High grade tumors	44	1	2.27%	4	9.09%	20	45.45%	19	43.18%	< 0.0001
Low grade tumors	22	7	31.81%	10	45.45%	5	22.72%	0	0	
Osteosarcoma	26	3	11.53%	5	19.23%	8	30.76%	10	38.46%	0.433
Ewing's sarcoma	26	6	23.07%	8	30.76%	9	34.61%	3	11.53%	
Chondrosarcoma	26	4	15.38%	8	30.76%	8	30.76%	6	23.07%	
Osteochondroma	24	12	50%	11	45.83%	1	4.16%	0	0	0.871
Giant cell tumor	24	13	54.16%	9	37.5%	2	8.33%	0	0	
Exostosis	24	10	41.66%	12	50%	2	8.33%	0	0	

^a^The expression pattern was determined based on the staining intensity of β-catenin antibody that is described in “Material and methods”.

## Supplementary Information

Supplementary Tables.

## Data Availability

All data generated or analyzed during this study and supporting our findings are included and can be found in the manuscript. The raw data can be provided by the corresponding author on reasonable request.

## Abbreviations

NKD2: Naked cuticle homolog 2
PBMC: Peripheral blood mononuclear cell
PBS: Phosphate-buffered saline
OCT: Optimal cutting temperature
ANOVA: One-way analysis of variance
SEM: Standard error mean
EMT: Epithelial-mesenchymal transition
FOXM1: Forkhead box protein M1
GCT: Giant cell tumor
H&E staining: Hematoxylin and Eosin staining
GSK: Glycogen synthase kinase
