# Supplementary material for: Expression pattern and clinical significance of β-catenin gene and protein in patients with primary malignant and benign bone tumors
Source: Sci Rep. 2022 Jun 8;12:9488. doi: 10.1038/s41598-022-13685-1 (PMC9177768; doi:10.1038/s41598-022-13685-1)
Supplement: Supplementary file 1 — Supplementary Tables. [file 41598_2022_13685_MOESM1_ESM.docx]

**Supplementary files (Tables):**

**Table S1**: The correlation of β-catenin expression in osteosarcoma with gene expression level in tumor tissue, PBMC, protein expression level, tumor size, tumor Grade, Huvos Grading, metastasis status and patient’s age.

| variable | Spearman's rho | **Tumor gene expression** | **PBMC gene expression** | **Tumor protein expression** |
| --- | --- | --- | --- | --- |
| **Tumor gene expression** | Correlation  P-value | 1  0.000 | 0.456  0.019* | 0.639  0.001** |
| **PBMC gene expression** | Correlation  P-value | 0.456  0.019* | 1  0.000 | 0.56  0.004** |
| **Tumor protein expression** | Correlation  P-value | 0.639  0.001** | 0.56  0.004** | 1  0.000 |
| **Tumor size** | Correlation  P-value | 0.467  0.03* | 0.411  0.058 | 0.409  0.073 |
| **Tumor Grade** | Correlation  P-value | 0.509  0.013* | 0.549  0.007** | 0.664  0.001** |
| **Huvos grade** | Correlation  P-value | -0.645  0.003** | -0.410  0.081 | -0.731  0.001** |
| **Metastasis** | Correlation  P-value | 0.548  0.004** | 0.448  0.022* | 0.676  <0.0001*** |
| **Age** | Correlation  P-value | 0.074  0.719 | -0.054  0.79 | 0.264  0.212 |
| **Tumor recurrence** | Correlation  P-value | 0.689  <0.0001**** | 0.517  0.007** | 0.761  <0.0001**** |

**Table S2**: The correlation of β-catenin expression in chondrosarcoma with gene expression level in tumor tissue, PBMC, protein expression level, tumor size, tumor Grade, Huvos Grading, metastasis status and patient’s age.

| variable | Spearman's rho | **Tumor gene expression** | **PBMC gene expression** | **Tumor protein expression** |
| --- | --- | --- | --- | --- |
| **Tumor gene expression** | Correlation  P-value | 1  0.000 | 0.34  0.088 | 0.385  0.052 |
| **PBMC gene expression** | Correlation  P-value | 0.34  0.088 | 1  0.000 | 0.526  0.006** |
| **Tumor protein expression** | Correlation  P-value | 0.385  0.052 | 0.526  0.006** | 1  0.000 |
| **Tumor size** | Correlation  P-value | 0.459  0.032* | 0.511  0.015* | 0.783  <0.0001**** |
| **Tumor Grade** | Correlation  P-value | 0.576  0.005** | 0.49  0.021* | 0.788  <0.0001**** |
| **Metastasis** | Correlation  P-value | 0.491  0.011* | 0.48  0.013* | 0.772  <0.0001**** |
| **Age** | Correlation  P-value | 0.132  0.52 | 0.559  0.003** | 0.285  0.159 |

**Table S3**: The correlation of β-catenin expression in Ewing’s Sarcoma with gene expression level in tumor tissue, PBMC, protein expression level, tumor size, tumor Grade, Huvos Grading, metastasis status and patient’s age.

| variable | Spearman's rho | **Tumor gene expression** | **PBMC gene expression** | **Tumor protein expression** |
| --- | --- | --- | --- | --- |
| **Tumor gene expression** | Correlation  P-value | 1  0.000 | 0.653  <0.0001**** | 0.704  <0.0001**** |
| **PBMC gene expression** | Correlation  P-value | 0.653  <0.0001**** | 1  0.000 | 0.840  <0.0001**** |
| **Tumor protein expression** | Correlation  P-value | 0.704  <0.0001**** | 0.840  <0.0001**** | 1  0.000 |
| **Tumor size** | Correlation  P-value | 0.699  <0.0001**** | 0.509  0.018* | 0.518  0.016* |
| **Tumor Grade** | Correlation  P-value | 0.646  0.002** | 0.379  0.09 | 0.583  0.006** |
| **Huvos grade** | Correlation  P-value | -0.454  0.098 | -0.661  0.010* | -0.479  0.102 |
| **Metastasis** | Correlation  P-value | 0.59  0.001** | 0.691  <0.0001**** | 0.633  0.001** |
| **Age** | Correlation  P-value | 0.261  0.198 | 0.246  0.227 | 0.320  0.110 |
| **Tumor recurrence** | Correlation  P-value | 0.572  0.002** | 0.758  <0.0001**** | 0.682  <0.0001**** |

**Table S4**: The correlation of β-catenin expression in osteochondroma tumors with gene expression level in tumor tissue, PBMC, protein expression level, tumor size and patient’s age.

| variable | Spearman's rho | **Tumor gene expression** | **PBMC gene expression** | **Tumor protein expression** |
| --- | --- | --- | --- | --- |
| **Tumor gene expression** | Correlation  P-value | 1  0.000 | 0.047  0.829 | -0.019  0.933 |
| **PBMC gene expression** | Correlation  P-value | 0.047  0.82 | 1  0.000 | 0.383  0.079 |
| **Tumor protein expression** | Correlation  P-value | -0.019  0.93 | 0.38  0.079 | 1  0.000 |
| **Tumor size** | Correlation  P-value | 0.098  0.68 | 0.32  0.17 | 0.515  0.029* |
| **Age** | Correlation  P-value | 0.26  0.219 | 0.261  0.218 | 0.071  0.75 |

**Table S5**: The correlation of β-catenin expression in Giant cell tumors with gene expression level in tumor tissue, PBMC, protein expression level, tumor size and patient’s age.

| variable | Spearman's rho | **Tumor gene expression** | **PBMC gene expression** | **Tumor protein expression** |
| --- | --- | --- | --- | --- |
| **Tumor gene expression** | Correlation  P-value | 1  0.000 | 0.11  0.607 | 0.523  0.009** |
| **PBMC gene expression** | Correlation  P-value | 0.11  0.6 | 1  0.000 | 0.06  0.78 |
| **Tumor protein expression** | Correlation  P-value | 0.523  0.009** | 0.06  0.78 | 1  0.000 |
| **Tumor size** | Correlation  P-value | 0.55  0.015* | 0.49  0.03* | 0.662  0.002** |
| **Age** | Correlation  P-value | -0.08  0.7 | -0.2  0.18 | -0.07  0.73 |

**Table S6**: The correlation of β-catenin expression in exostosis tumors with gene expression level in tumor tissue, PBMC, protein expression level, tumor size and patient’s age.

| variable | Spearman's rho | **Tumor gene expression** | **PBMC gene expression** | **Tumor protein expression** |
| --- | --- | --- | --- | --- |
| **Tumor gene expression** | Correlation  P-value | 1  0.000 | 0.089  068 | 0.404  0.05 |
| **PBMC gene expression** | Correlation  P-value | 0.09  0.68 | 1  0.000 | 0.061  0.778 |
| **Tumor protein expression** | Correlation  P-value | 0.404  0.05 | 0.061  0.77 | 1  0.000 |
| **Tumor size** | Correlation  P-value | 0.36  0.1 | 0.12  0.59 | 0.247  0.28 |
| **Age** | Correlation  P-value | -0.243  0.25 | 0.045  0.83 | -0.142  0.5 |

**Table S7**: The regression model of β-catenin tumor expression in patients with bone cancer

| **Variables** |  | **Metastasis** | | | |  | | **Grade** | | |
| --- | --- | --- | --- | --- | --- | --- | --- | --- | --- | --- |
|  | **P-value** | **B** | **OR** | **95% C.I for OR** | | **P-value** | **B** | **OR** | **95% C.I for OR** | |
|  |  |  |  | **lower** | **upper** |  |  |  | **lower** | **upper** |
| **Tumor gene expression** | 0.14 | 0.44 | 1.55 | 0.865 | 2.794 | **0.023*** | 0.841 | 2.319 | 1.123 | 4.790 |
| **PBMC gene expression** | 0.548 | 0.01 | 1.01 | 0.979 | 1.042 | 0.286 | 0.032 | 1.032 | 0.974 | 1.095 |
| **Tumor protein expression** | **0.005*** | 1.657 | 5.246 | 1.659 | 16.586 | **0.012*** | 1.909 | 6.749 | 1.532 | 29.734 |
| **age** | 0.08 | 0.667 | 1.948 | 0.924 | 4.106 | 0.259 | -0.604 | 0.547 | 0.191 | 1.561 |
| **gender** | 0.902 | 0.087 | 1.09 | 0.272 | 4.380 | 0.203 | -1.171 | 0.310 | 0.051 | 1.883 |
